# Citron Rho-Interacting Serine/Threonine Kinase Promotes HIF1a-CypA Signaling and Growth of Human Pancreatic Adenocarcinoma

**DOI:** 10.1155/2020/9210891

**Published:** 2020-02-22

**Authors:** Lin Cong, Zhile Bai, Yang Du, Yong Cheng

**Affiliations:** ^1^Department of General Surgery, Peking Union Medical College Hospital, Chinese Academy of Medical Sciences & Peking Union Medical College, Beijing 100730, China; ^2^Key Laboratory of Ethnomedicine for Ministry of Education, Center on Translational Neuroscience, College of Life and Environmental Sciences, Minzu University of China, Beijing, China

## Abstract

In human pancreatic ductal adenocarcinoma (PDAC), the cyclophilin A (CypA) is overexpressed and promotes the development of PDAC. However, the mechanism underlying cyclophilin A expression remains elusive. Here, we reported that the citron Rho-interacting serine/threonine kinase (CIT) promotes the HIF1a-CypA signaling and growth of PDAC cells. CIT expression was higher in PDAC cells compared with the normal epithelial cells, and clinical data showed that CIT was overexpressed in PDAC tissues and high expression of CIT predicted poor overall and disease-free survival. In PDAC cells, knockdown of CIT expression repressed the rate of proliferation and capacity of colony formation, which were accomplished with an increased percentage of apoptotic cells and cell cycle arrest. The knockdown of CIT in PDAC cells reduced the expression of CypA while overexpression of CIT promoted the expression of CypA. We observed that the effects of CIT on the expression of CypA relied on the transcriptional factor HIF1a, which was previously reported to transcriptionally activate the expression of CypA in PDAC cells. Furthermore, the effects of CIT on apoptosis, cell cycle, proliferation, and colony formation of PDAC cells relied on its role in the regulation of CypA expression. Collectively, our data showed that CIT promoted the activation of HIF1-CypA signaling and enhanced the growth of PDAC cells.

## 1. Introduction

Pancreatic ductal adenocarcinoma (PDAC) is one of the leading causes of cancer-related death in men throughout the world. This fact results from the limited knowledge-based treatment strategies [1]. During the past two decades, the high-throughput genome-scale screening and GWAS studies have identified dozens of risk mutations and oncogenic genes that are essentially correlated with the initiation and progression of this disease [2–5]. Also, recent immune landscape analysis has identified some rate-limiting immune checkpoints for the development and therapeutic resistance for PDAC [6–9]. However, the current therapeutic choice for PDAC is still limited, and the information for individual risk gene or protein is far from clear, which requires us to have a better understanding of the mechanism underlying this malignancy.

Cyclophilin A (CypA), one of the members of the immunophilin family, is ubiquitously distributed. This protein is a peptidylprolyl cis-trans isomerase, which functions to modulate protein processing (folding and trafficking). CypA is secreted by cells in response to inflammatory stimuli. *Via* its receptor CD147, the secreted CypA binds to the cell surface and induces the production and secretion of inflammatory cytokines [10]. CypA has various functions in inflammatory conditions and diseases, including viral infections, cardiovascular diseases, neurodegeneration, aging, rheumatoid arthritis, periodontitis, sepsis, and asthma [11]. The roles of CypA in the development of human cancer have been widely investigated. For example, CypA is upregulated in the majority of patients with liver fluke-associated cholangiocarcinoma (CCA) and favors the growth advantage in CCA cells [12]. In glioma, CypA enhances the stemness, self-renewal, and radioresistance of glioma-initiating cells *via* the Wnt/*β*-catenin signaling pathway [13]. In human multiple myeloma, bone marrow endothelial cells secreted extracellular CyPA (eCyPA), which bounds its receptor, CD147, on multiple myeloma cells to facilitate the colonization and proliferation of multiple myeloma cells [14]. A clinical study revealed that high expression of CypA is correlated with cell apoptosis, metastasis, tumor invasion, and chemoresistance in pancreatic adenocarcinoma [15]. However, the mechanism underlying CypA overexpression in human PDAC remains unknown.

Citron Rho-Interacting Serine/Threonine Kinase (CIT) is a serine/threonine kinase, which functions as a key component of the midbody [16–18], and is critically essential for cell division [16, 19–21], and participates in cytokinesis [22–26]. Mutations in CIT cause severe primary microcephaly in humans [23, 25, 27–29]. Recently, the role of CIT in human cancer has been identified. For instance, CIT promotes the development of cancer, and high expression of CIT predicts poor outcomes in human colon cancer [20]. In multiple myeloma, CIT expression is upregulated and correlated with patients' worse survival [22]. The overexpression of CIT in human ovarian and hepatocellular carcinoma has also been observed [30, 31]. RNA interference targeting CIT can significantly inhibit the proliferation of ovarian cancer cells and hepatocellular carcinoma cells [30, 31]. In medulloblastoma, inactivation of Citron kinase inhibits medulloblastoma progression by inducing apoptosis and cell senescence [32]. However, the functions of CIT in human PDAC remain unknown.

In this work, we studied the mechanism underlying CypA upregulation in PDAC cells. The CIT kinase promotes the expression of CypA in PDAC cells in a HIF1-dependent manner. CIT is overexpressed in human PDAC cells and correlates with the expression of CypA. High expression of the CIT gene predicts unfavorable overall and disease-free survival in patients with PDAC. In addition, our findings show that CIT promotes the growth and represses apoptosis of PDAC cells, which relies on the regulation of CIT on CypA expression.

## 2. Materials and Methods

### 2.1. Clinical Data of PDAC Patients

The CIT expression and survival data of patients with PDAC were obtained from the TGCA database. The analysis was performed with the online tool GEPIA (http://gepia.cancer-pku.cn/). 171 cases of normal controls and 179 cases of PDAC patients were included in the analysis of gene expression. 178 cases of PDAC patients with full prognostic data were included in the analysis of survival. The 178 patients were divided into *CIT* low group (*n* = 89) and *CIT* high group (*n* = 89) by the median expression value.

### 2.2. PDAC Cancer Cells

The normal human pancreatic duct epithelial cell line (H6C7) was obtained from the Kerafast (ECA001-FP). The PDAC cancer cells Capan-1, BxPC-3, AsPC-2, MiPaCa II, and PANC-1 were purchased from the Global Bioresource Center (ATCC). All these lines of cells were cultured in the DMEM medium (Hyclone) supplement with 10% Fetal Bovine Serum (Gibco), 1% Antibiotic-Antimycotic (Gibco).

### 2.3. Lentivirus Packaging and Gene Knockdown or Overexpression

For *CIT* and *CypA* knockdown and *CIT* overexpression, the lentivirus system was applied. The *CIT* and *CypA* shRNA lentivirus were purchased from Sigma. For CIT or *CypA* overexpression, the human *CIT* or *CypA* expression construct was cloned into the pLJM1-EGFP plasmid. Then, the pLJM1-EGFP-*CIT* plasmid was cotransfected with psPAX2 and pMD2.G into HEK293T cells for lentivirus packaging. Capan-1 and BxPC-3 cells were infected with lentivirus and selected with puromycin (1 *μ*M) for 48 hours. The targeting sequences of the shRNAs are listed below:

   shCIT-1# 5′-GCCAATAAACTTGCAGCAAAT-3′
  shCIT-2# 5′-CGGAAGTATTCCGACACCATA-3′
  shCypA 5′-ACTTCACCAACTGCCACACCAA-3′

### 2.4. Quantitative Real-Time PCR (qPCR)

For RNA isolation, the cultured cells were extracted with TRIZOL reagent (Invitrogen). Then the genomic DNA was depleted and the first-strand cDNA was synthesized with the RevertAid First-Strand cDNA Synthesis Kit (ThermoFisher). Next, the qPCR experiment was carried out to analyze the expression of target genes with the QuantiTect SYBR® Green PCR Kit (QIAGEN) and the following primers:

  Human *CIT* sense: 5′-ATATGGAGCGCGGAATCCTTT-3′
  Human *CIT* antisense: 5′TCAGCTATGGTGTCGGAATACT3′
  Human *CypA* sense: 5′-CCCACCGTGTTCTTCGACATT-3′
  Human *CypA* antisense: 5′-GGACCCGTATGCTTTAGGATGA-3′
  Human *HIF1A* sense: 5′-TTCCCGACTAGGCCCATTC-3′
  Human *HIF1A* antisense: 5′- CAGGTATTCAAGGTCCCATTTCA-3′
  Human *GAPDH* sense: 5′-TGTGGGCATCAATGGATTTGG-3′
  Human *GAPDH* antisense: 5′- ACACCATGTATTCCGGGTCAAT-3′

### 2.5. Western Blot

To prepare protein for western blot, the cultured cells were lysed with RIPA reagent (Millipore) supplied with protease inhibitor cocktail (Biomake). Then the same amount of total protein was subjected to SDS-PAGE separation and incubation with primary antibodies at room temperature for one hour, followed by secondary antibody incubation at room temperature for two hours. The anti-CIT antibody was purchased from Abcam (ab110897), an anti-CYPA antibody from Santa Cruz Biotechnology (sc-134310), an anti-GAPDH antibody from ProteinTech (60004-1-Ig), an anti-HIF1A antibody from ProteinTech (20960-1-AP).

### 2.6. Cell Proliferation Assay

For cell proliferation experiment, the transduced Capan-1 and BxPC-3 cells were plated into 96-well plates and cultured in DMEM medium. Cell number was monitored at day 0 to day 3 with the Cell Counting Kit-8 (CCK-8) kit (C0038) from Beyotime Biotechnology.

### 2.7. Colony Formation Assay

For colony formation assay, the transduced Capan-1 and BxPC-3 cells were plated in 6-well plates and cultured in full DMEM medium for 14 days, the medium was replaced every other two days. Then the colonies were fixed with Colony fixation solution and stained with Crystal violet solution (Beyotime Biotechnology), cell number per well were calculated.

### 2.8. Apoptosis and Cell Cycle Evaluation

Capan-1 and BxPC3 cells were infected with lentivirus carrying shRNA targeting *CIT* or *CypA* or control shRNA for 48 hours. Then cells were fixed and stained with Annexin V-FITC kit (Beyotime Biotechnology) and analyzed with flow cytometry to test cell apoptosis. The percentage of apoptotic cells was calculated. The cell cycle was analyzed with Cell Cycle Assay Kit (Abcam, ab112116) by flow cytometry. Ki67 expression was analyzed with an APC-conjugated anti-Ki67 antibody (ThermoFisher, 17-5698-82) by flow cytometry.

### 2.9. Statistical Analysis

In this study, all the values were pressed as mean ± SD of at least three independent repeats. For data of two groups, Student's *t*-test was applied to analyze the difference. For data of more than two groups, one-way or two-way ANOVA test followed by Bonferroni's multiple comparisons *post hoc* test was performed to analyze the difference. For the linear regression analysis, the Pearson correlation test was applied. For survival analysis, the Log-rank (Mantel-Cox) test was applied.

## 3. Results

### 3.1. CIT Is Overexpressed in PDAC Tissues and Predicts Poor Survival

**Figure 1:**
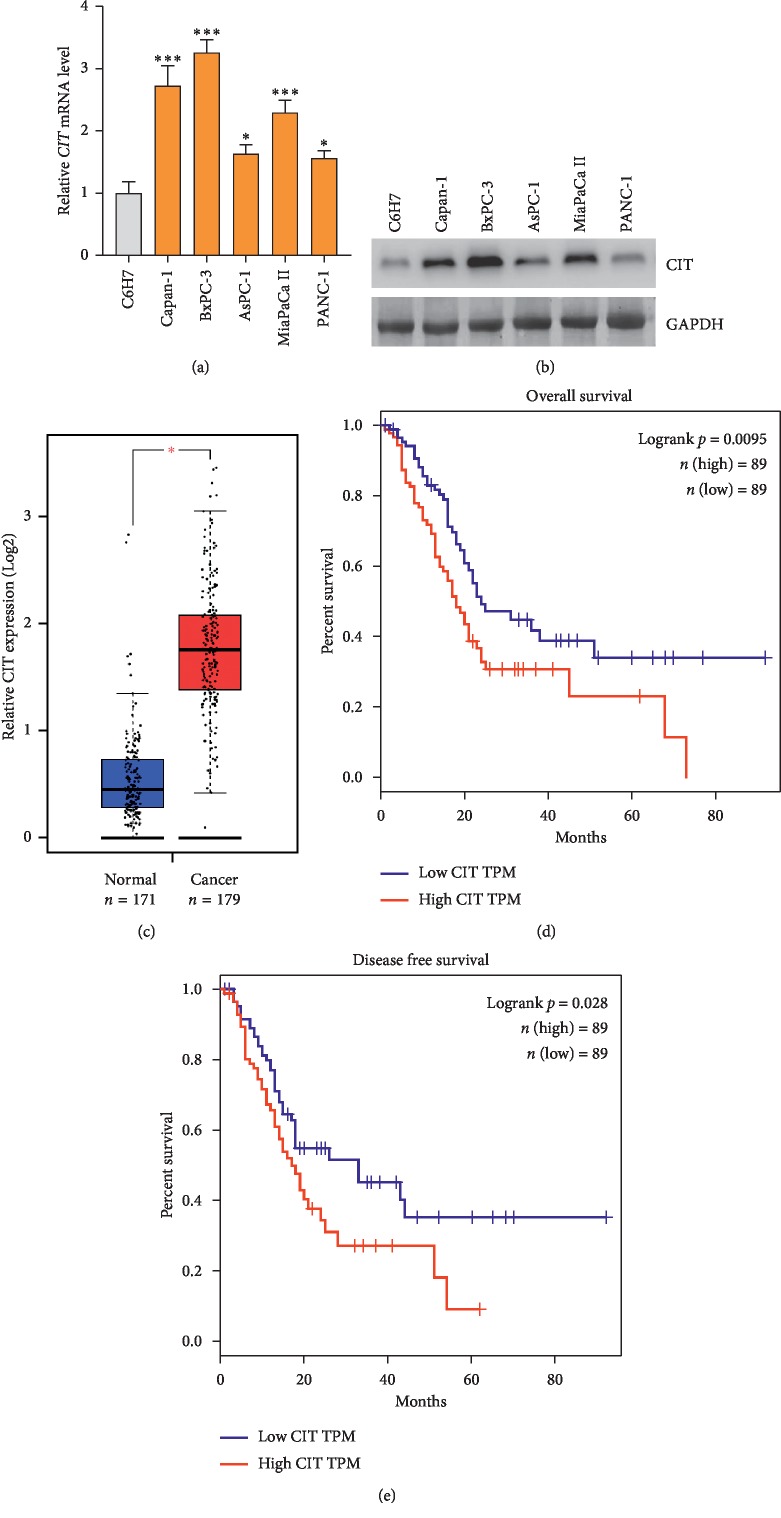
*CIT* is overexpressed in human PDAC and predicts poor survival. (a) *CIT* mRNA is overexpressed in PDAC cells. qPCR was performed to analyze the expression of *CIT* in five PDAC cell lines (Capan-1, BxPC-3, AsPC-1, MiaPaCa II, and PANC-1) and one normal human pancreatic duct epithelial cell line (H6C7). ^*∗*^*p* < 0.05, ^*∗∗∗*^*p* < 0.001*vs.* H6C7. (b) *CIT* protein level is overexpressed in PDAC cells. Western blot was performed to analyze the expression of *CIT* in five PDAC cell lines (Capan-1, BxPC-3, AsPC-1, MiaPaCa II, and PANC-1) and one normal human pancreatic duct epithelial cell line (H6C7). (c) *CIT* expression is overexpressed in PDAC tissues. The expression of CIT in PDAC was analyzed using the data from the TGCA database with the tool GEPIA (http://gepia.cancer-pku.cn). 171 normal tissues and 179 PDAC tissues were involved in the analysis. ^*∗*^*p* < 0.01. (d) *CIT* high expression predicts poor overall survival. The patients were cut into *CIT* high expression (*n* = 89) and *CIT* low expression (*n* = 89) groups by the median expression value. Log-rank (Mantel-Cox) test was applied for survival analysis. (e) *CIT* high expression predicts poor disease-free survival. Log-rank (Mantel-Cox) test was applied for survival analysis.

To investigate the function of CIT in the development and progress of human PDAC, we first examined the expression pattern of CIT in human PDAC cell lines and tissues. We analyzed the mRNA and protein levels of CIT in five PDAC cell lines (Capan-1, BxPC-3, AsPC-1, MiaPaCa II, and PANC-1) and one normal control cell line, the human pancreatic duct epithelial cell line (H6C7). The qPCR and western blot results showed that the expression of CIT in PDAC cell lines was much higher than that in the H6C7 cells (Figures 1(a) and 1(b)). We next analyzed the expression of CIT in human PDAC tissues using the data from the TGCA database with the online tool GEPIA (http://gepia.cancer-pku.cn/). 171 normal control tissues and 179 PDAC tissues were involved in the analysis. The results showed that the expression of CIT was significantly upregulated in the PDAC tissues compared with the noncancer control tissues (Figure 1(c)). Finally, we performed survival analysis by dividing the patients into CIT high expression (*n* = 89) and CIT low expression (*n* = 89). The results showed that high expression of CIT in PDAC tissues predicted unfavorable overall (*p*=0.0095) and disease-free (*p*=0.028) survival (Figures 1(d) and 1(e)). Collectively, these results demonstrated that CIT was overexpressed in human PDAC tissues and CIT high expression predicted poor overall and disease-free survival in patients with PDAC.

### 3.2. CIT Promotes the Growth of PDAC Cells

**Figure 2:**
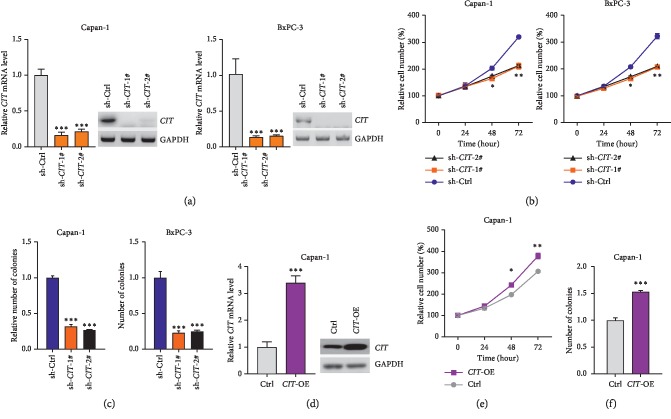
*CIT* promotes the growth of PDAC cells. (a) *CIT* knockdown in Capan-1 and BxPC-3 cells. Capan-1 and BxPC-3 cells were infected with lentivirus carrying sh-Ctrl or sh-*CIT* for 24 hours, and then qPCR and western blot were applied to analyze the expression of *CIT*. ^*∗∗∗*^*p* < 0.001*vs.* sh-Ctrl. (b) *CIT* knockdown reduces the rate of proliferation of PDAC cells. Capan-1 and BxPC-3 cells with stable *CIT* knockdown were used for cell proliferation experiments. ^*∗*^*p* < 0.05, ^*∗∗*^*p* < 0.01*vs.* sh-Ctrl. (c) *CIT* knockdown reduces the ability of colony formation in PDAC cells. Capan-1 and BxPC-3 cells with stable *CIT* knockdown were used for colony formation experiment, the colonies were cultured for 14 days before final analysis. ^*∗∗∗*^*p* < 0.001*vs.* sh-Ctrl. (d) *CIT* overexpression in Capan-1 cells. Capan-1 cells were infected with lentivirus overexpressing *CIT* (*CIT*-OE) and control construct for 24 hours, then qPCR and western blot were applied to analyze the expression of *CIT*. ^*∗∗∗*^*p* < 0.001*vs.* Ctrl. (e) *CIT* overexpression promotes the rate of proliferation of PDAC cells. Capan-1 cells with stable *CIT* overexpression (*CIT*-OE) were applied to cell proliferation experiments. ^*∗*^*p* < 0.05, ^*∗∗*^*p* < 0.01*vs.* Ctrl. (f) *CIT* overexpression enhances the capacity of colony formation of PDAC cells. Capan-1 cells with stable *CIT* overexpression were used for colony formation experiment, the colonies were cultured for 14 days before final analysis. ^*∗∗∗*^*p* < 0.001*vs.* Ctrl.

We next analyzed whether CIT indeed participated in the cellular behavior of PDAC cells. We selected Capan-1 and BxPC-3 cells with stable CIT knockdown or overexpression (Figure 2(a)), and the cells were subjected to cellular proliferation and colony formation experiments. The results showed that CIT knockdown significantly decreased the rate of proliferation of Capan-1 and BxPC-3 cells (Figure 2(b)). Additionally, we observed that CIT knockdown reduced the capacity of colony formation of Capan-1 and BxPC-3 cells with a decreased number of colonies (Figure 2(c). By contrast, overexpression of CIT promoted the growth of Capan-1 cells because of a higher proliferation rate and an increased number of colonies were observed (Figures 2(d)–2(f)). Therefore, CIT was an essential contributor to the growth of PDAC cells.

### 3.3. CIT Regulates Apoptosis and Cell Cycle of PDAC Cells

**Figure 3:**
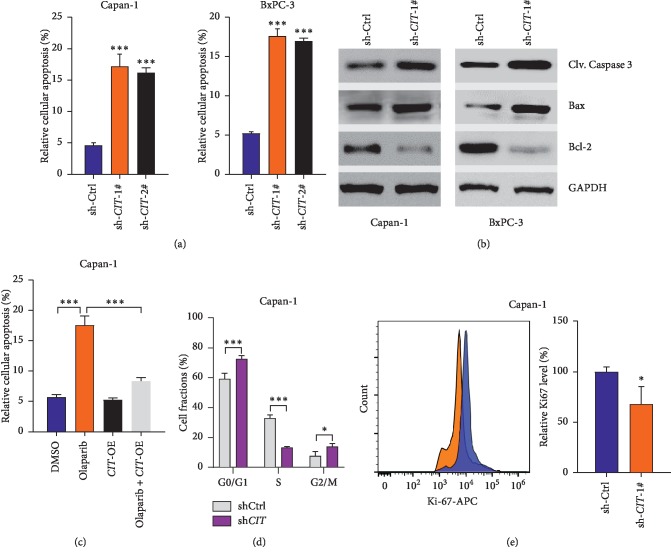
CIT regulates apoptosis and cell cycle of PDAC cells. (a) *CIT* knockdown induces apoptosis in PDAC cells. Capan-1 and BxPC-3 cells were infected with lentivirus carrying sh-Ctrl or sh-*CIT* for 48 hours, then apoptosis of the cells was monitored. ^*∗∗∗*^*p* < 0.001 vs. sh-Ctrl. (b) *CIT* knockdown activates apoptotic activators in PDAC cells. Capan-1 and BxPC-3 cells were infected with lentivirus carrying sh-Ctrl or sh-*CIT* for 24 hours, and then western blot was applied to analyze apoptotic effectors. (c) *CIT* overexpression represses olaparib-induced apoptosis. Capan-1 with/without CIT overexpression was treated with olaparib (1 *μ*M) for 24 hours, then apoptosis of the cells was monitored. ^*∗∗∗*^*p* < 0.001. (d) *CIT* knockdown induces cell cycle arrest. Capan-1 cells were infected with lentivirus carrying sh-Ctrl or sh-*CIT* for 48 hours, and then the cell cycle of the cells was monitored with flow cytometry. ^*∗*^*p* < 0.05, ^*∗∗∗*^*p* < 0.001*vs.* sh-Ctrl. (e) *CIT* knockdown reduces Ki67 expression. Capan-1 cells were infected with lentivirus carrying sh-Ctrl or sh-*CIT* for 48 hours, and then Ki67 expression of the cells was monitored with flow cytometry. ^*∗*^*p* < 0.05*vs.* sh-Ctrl.

In addition, we also analyzed whether the effects of CIT on PDAC was attributed to its effect on cell survival. We observed that CIT knockdown significantly induced apoptosis of Capan-1 and BxPC-3 cells (Figure 3(a)). Western blot analysis also revealed that CIT knockdown activated the proapoptotic activators such as Caspase 3 and Bax whereas the anti-apoptotic Bcl-2 was repressed by CIT knockdown (Figure 3(b)). To further test whether CIT protected cells against apoptosis, we overexpressed CIT in Capan-1 cells and induced apoptosis with olaparib. The results showed that CIT overexpression repressed olaparib-induced apoptosis of Capan-1 cells (Figure 3(c)). To test other mechanisms underlying repressed cell growth, cell cycle and cell proliferation were analyzed. We observed that CIT knockdown induced cell cycle arrest with an increased percentage at G0/G1 and G2/M phases and reduced percentage at S phase, indicating that CIT knockdown induces cytokinesis failure (Figure 3(d)). The reduced proliferation was also evidenced by decreased expression of Ki67 in Capan-1 cells with CIT knockdown (Figure 3(e)). Taken together, these findings indicate that CIT regulates cell growth in PDAC via a diverse mechanism.

### 3.4. CIT Regulates the Expression of CypA

**Figure 4:**
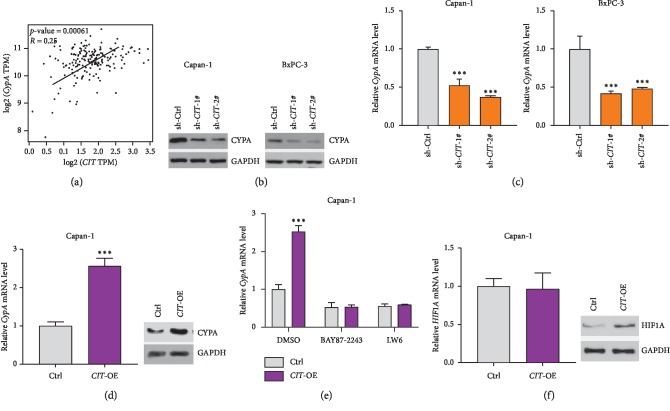
*CIT* promotes the expression of *CypA* in a HIF1-dependent manner. (a) *CIT* expression is correlated with *CypA* in PDAC tissues. The expressions of *CIT* and *CypA* in PDAC tissues were analyzed using the data from the TGCA database with the web tool GEPIA (http://gepia.cancer-pku.cn). (b) *CIT* knockdown reduces the protein level of *CypA*. Capan-1 and BxPC-3 cells were infected with lentivirus carrying sh-Ctrl or sh-*CIT* for 24 hours, and then western blot was applied to analyze the expression of *CypA*. (c) *CIT* knockdown reduces the mRNA level of *CypA.* Capan-1 and BxPC-3 cells were infected with lentivirus carrying sh-Ctrl or sh-*CIT* for 24 hours, then qPCR was applied to analyze the expression of *CypA.*^*∗∗∗*^*p* < 0.001*vs.* sh-Ctrl. (d) *CIT* overexpression increases the level of *CypA*. Capan-1 cells were infected with lentivirus carrying Ctrl or *CIT* expressing *construct* for 24 hours, then qPCR and western blot were applied to analyze the expression of *CypA*. ^*∗∗∗*^*p* < 0.001*vs.* Ctrl. (e) HIF-1 inhibition blocks the effects of CIT on CypA expression. Capan-1 cells were infected with lentivirus carrying Ctrl or *CIT* expressing *construct* for 24 hours in the presence of HIF-1 inhibitors BAY 87-2243 (1 *μ*M), LW 6 (10 *μ*M), or DMSO, and then qPCR was applied to analyze the expression of *CypA*. ^*∗∗∗*^*p* < 0.001*vs.* Ctrl + DMSO. (f) *CIT* overexpression increases the protein level of HIF1A. Capan-1 cells were infected with lentivirus carrying Ctrl or *CIT* expressing *construct* for 24 hours, then qPCR and western blot were applied to analyze the expression of HIF1A.

A clinical study revealed that high expression of CypA is correlated with cell hyperproliferation, apoptosis, and chemoresistance in PDAC [15]. To investigate whether the CIT could act as a regulator of CypA expression, we first analyzed the correlation between CIT and CypA in human PDAC tissues using the data from the TGCA database with the online tool GEPIA (http://gepia.cancer-pku.cn/). The Pearson correlation analysis implicated that the expression of CypA was significantly and positively correlated with the expression of CIT (*R* = 0.25, *p*=0.00061, Figure 4(a)). We observed that the expression of the mRNA and protein of CypA was remarkedly reduced in Capan-1 and BxPC-3 cells with CIT knockdown (Figures 4(b) and 4(c)). Next, we overexpressed the expression of CIT in Capan-1 cells with lentivirus carrying CIT ORF (Figure 4(d)). Consistently, the mRNA and protein levels of CypA were increased by overexpressing CIT in the Capan-1 cells (Figure 4(e)). These results implicated that the CIT could promote the expression of CypA at the transcriptional level.

### 3.5. HIF1 Is Involved in the Regulation of CypA by CIT

We next asked how CIT regulated the expression of CypA in PDAC cells. A previous report showed that the hypoxia-responsive transcriptional factor HIF1*α* could directly bind to the hypoxia response element at the promoter of the *CypA* gene and activated the expression of *CypA* [15]. We tested whether HIF1*α* was involved in the regulation of CypA by CIT. The Capan-1 cells with or without CIT overexpression were treated with two HIF1 inhibitors, BAY 87-2243, and LW 6 or DMSO. The results showed that HIF1 inhibition indeed reduced the mRNA level of CypA. However, CIT overexpression was unable to activate the expression of the CypA gene when HIF1 was inhibited by BAY 87-2243 and LW 6 (Figure 4(e)). We also analyzed the effects of CIT on HIF1a expression, and the results showed that CIT overexpression promoted the protein level of HIF1A but did not affect its mRNA expression, implicating that CIT may regulate HIF1A stability (Figure 4(f)). Taken together, our results demonstrated that CIT promoted the expression of CypA in a HIF1-dependent manner in human PDAC cells, implicating that CIT may have a function in human PDAC.

### 3.6. CypA Contributes to the Effects of CIT on Growth and Apoptosis of PDAC Cells

**Figure 5:**
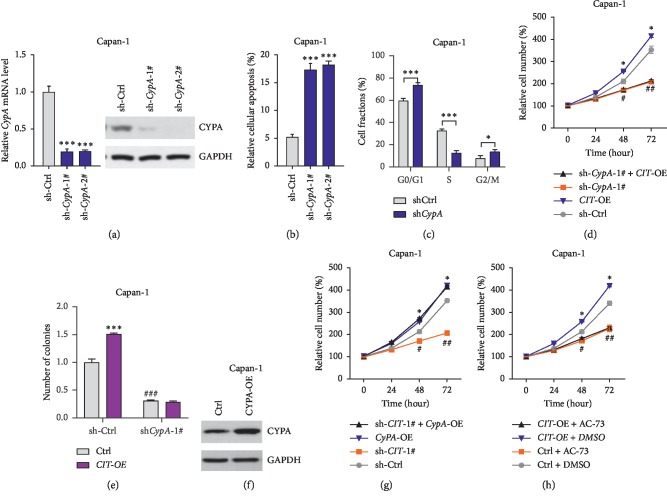
*CypA c*ontributes to the function of *CIT* in regulating growth and apoptosis of PDAC cells. (a) *CypA* knockdown in Capan-1 cells. Capan-1 cells were infected with lentivirus carrying sh-Ctrl or sh-*CypA* for 24 hours, qPCR and western blot were used to analyze *CypA* expression. ^*∗∗∗*^*p* < 0.001. (b) *CypA* knockdown induces apoptosis of Capan-1 cells. Capan-1 cells were infected with lentivirus carrying sh-Ctrl or sh-*CIT* for 48 hours, and then apoptosis of the cells was monitored. ^*∗∗∗*^*p* < 0.001 vs. sh-Ctrl. (c) *CypA* knockdown induces cell cycle arrest in Capan-1 cells. Capan-1 cells were infected with lentivirus carrying sh-Ctrl or sh-*CIT* for 48 hours, and then the cell cycle was monitored. ^*∗*^*p* < 0.05, ^*∗∗∗*^*p* < 0.001. (d) *CypA* knockdown blocks the effects of *CIT* on the proliferation of Capan-1 cells. Capan-1 with stable *CIT* overexpression, *CypA* knockdown, or both were subjected to cell proliferation experiment. ^*∗*^*p* < 0.05*vs.* sh-Ctrl; ^#^*p* < 0.05, ^##^*p* < 0.01*vs.* sh-Ctrl. (e) *CypA* knockdown blocks the effects of *CIT* on colony formation of Capan-1 cells. Capan-1 with stable *CIT* overexpression, *CypA* knockdown, or both were subjected to colony formation assay, and the colonies were cultured for 14 days before final analysis. ^*∗∗∗*^*p* < 0.001*vs.* sh-Ctrl; ^###^*p* < 0.01*vs.* sh-Ctrl. (f) *CypA* overexpression in Capan-1 cells. Capan-1 cells were infected with lentivirus overexpressing CypA or control construct for 24 hours. Then, CYPA expression was analyzed by western blot. (g) *CypA* overexpression rescues the effects of *CIT* on the proliferation of Capan-1 cells. Capan-1 with stable *CIT* knockdown, *CypA* overexpression, or both were subjected to cell proliferation experiment. ^*∗*^*p* < 0.05*vs.* sh-Ctrl; ^#^*p* < 0.05, ^##^*p* < 0.01*vs.* sh-Ctrl. (h) Inhibition of CypA receptor CD147 blocks the effects of *CIT* on the proliferation of Capan-1 cells. Capan-1 with/without stably *CIT* overexpression was treated with CD147 inhibitor AC-73 (5 *μ*M). ^*∗*^*p* < 0.05*vs.* Ctrl + DMSO; ^#^*p* < 0.05, ^##^*p* < 0.01*vs.* Ctrl + DMSO.

Finally, we analyzed whether the CypA, the downstream target of CIT, was critically involved in the function of CIT. We generated lentivirus carrying shRNA targeting CypA and the qPCR and western blot results showed CypA could be knocked down in Capan-1 cells (Figure 5(a)). The apoptosis experiments showed that CypA knockdown significantly increased the percentage of apoptotic cells in Capan-1 cells (Figure 5(b)) and induced cell cycle arrest with an increased percentage at G0/G1 and G2/M phases and reduced percentage at S phase, which was similar to the effects of CIT knockdown (Figure 5(c)). To further explore whether CypA contributes to the function of CIT, we knocked down CypA in Capan-1 cells with CIT overexpression. Significantly, the inhibition effects of CypA knockdown on proliferation and colony formation of Capan-1 cells were also observed (Figures 5(d) and 5(e)). Notably, CIT overexpression could not facilitate the proliferation or colony formation progress in Capan-1 cells with CypA knockdown (Figures 5(d) and 5(e)). By contrast, CypA overexpression rescued the rescued proliferation rate of Capan-1 cells with CIT knockdown (Figures 5(f) and 5(g)). We also inhibited CypA receptor CD147 with AC-73 and observed that AC-73 repressed the effects of CIT overexpression on cell proliferation of Capan-1 cells (Figure 5(h). These findings demonstrated the critical involvement of CypA in the CIT-mediated promotion of PDAC cell growth.

## 4. Discussion

Although CIT has been identified as a regulator for midbody and cell division, the roles of CIT in physiological and pathological progresses are not clear so far. CIT encodes citron and acts as an effector of the Rho signaling, which is necessary for cell division, especially in proliferating neuron progenitor cells, and postpartum brain development. Mutations in CIT cause severe primary microcephaly in humans [23, 29]. The roles of CIT have been recently uncovered. Overexpression of CIT has been observed in human ovarian cancer [30], hepatocellular carcinoma [31], colon cancer [20], and multiple myeloma [22]. In these types of cancer, high expression of CIT correlated with poor prognosis of patients [20, 22, 31]. However, the functions of CIT in other types of cancer remain unknown.

We observed that CIT was highly expressed in human PDAC cells and tissues. The Log-rank test revealed that a high expression of CIT was correlated with unfavorable overall and disease-free survival, which implicating that CIT may serve as a potential prognostic factor. We demonstrated that CIT promotes the growth and apoptotic resistance in PDAC cells in a CypA-dependent manner. Previously, studies have already identified CIT as a regulator of midbody and cell division, and nearly all studies on CIT functions in cancer focused the effects of CIT on cell cycle [20, 22]. Indeed, we observed that CIT promoted cell growth in PDAC. Notably, our data showed that CIT maintained the cellular survival of PDAC cells. Knockdown of CIT in PDAC cells induced apoptosis, cell cycle arrest, and cytokinesis failure. Therefore, CIT targets either cell cycle or survival to maintain the high proliferation rate of cancer cells.

CypA has been identified to participate in diverse types of cancer [10–14]. This fact implicates that CypA could be a potential target for the treatment of these cancers. In human PDAC, CypA was overexpressed and the high expression of this protein was correlated with worse overall and disease-free survival [15]. However, the mechanism by which CypA was regulated in human PDAC was not fully understood. Here in this study, we provide evidence that CIT acted as an upstream regulator of CypA expression. In human PDAC tissues, the expression of CIT was positively correlated with the expression of CypA. In PDAC cells, we showed that CIT knockdown reduced the expression level of CypA whereas CIT overexpression led to an opposite result. Interestingly, we observed that the upregulation of CypA by CIT relied on the transcriptional factor HIF1, which was previously reported to transcriptionally activated CyPA [30]. In addition, we observed that CIT overexpression promoted the expression of HIF1A protein but mRNA, implicating that CIT may regulate HIF1A stability. Taken together, the CIT-HIF signaling axis is an effective upstream regulator for CypA, which contributed to the function of CIT in human PDAC.

## 5. Conclusions

Here, we identified CIT as an oncogene-like protein in human PDAC. CIT activates the HIF1-CypA signaling and promotes the growth of human PDAC cells. In PDAC cells and tissues, CIT is overexpressed and CIT high expression predicts unfavorable overall and disease-free survival in patients with PDAC. CIT regulates proliferation and colony formation of PDAC cells partially through maintaining the survival of cells. Mechanism study implicated that CIT promotes the transcription of CypA in a HIF1-dependent manner and CypA is critically involved in the function of CIT in the regulation of PDAC cell growth. In conclusion, we identified that CIT acts as an upstream activator of the HIF1-CypA signaling and facilitates the growth of human PDAC. CIT may be a promising target for the treatment of PDAC in human patients.

## Data Availability

The data used to support the findings of this study are available from the corresponding author upon request.
